# Turner syndrome with Xp deletions and rare endometrial abnormalities: A case report

**DOI:** 10.1097/MD.0000000000027571

**Published:** 2021-11-19

**Authors:** Lei Liang, Libin Mei, Yingying Shi, Lingling Huang, Zhiying Su, Yu Zeng, Haijie Gao, Xuemei He, Hui Huang, Yanru Huang, Ping Li, Jing Chen

**Affiliations:** a Women and Children's Hospital, School of Medicine, Xiamen University, Xiamen, Fujian, China; b Xiamen Key Laboratory of Reproduction and Genetics, Xiamen, Fujian, China; c School of Public Health, Xiamen University, Xiamen, Fujian, China; d Reproductive Medicine Center, the First Affiliated Hospital of Xiamen University, Fujian, Xiamen, China.; e Research Group for Reproductive Medicine and IVF Laboratory, Department of Obstetrics and Gynecology, Cologne University, Cologne, Germany.

**Keywords:** endometrial abnormalities, ovarian hypoplasia, Turner syndrome, Xp deletion

## Abstract

**Rationale:**

Turner syndrome (TS) is a genetic disorder associated with abnormalities of the X chromosome related to ovarian function, but whether it is associated with endometrial abnormalities is still not clear.

**Patient concerns:**

We report the case of a 26-year-old Han Chinese woman with TS and Xp11.2 deletion, presenting with short final stature, ovarian hypofunction, unexplained cystic dilatation of the entire endometrium, and endometrial thickening.

**Diagnoses:**

The patient was diagnosed with chromosome Xp11.2 deletion through cytogenetic analysis and ultrasonic and endometrial pathology.

**Interventions:**

The patient was treated with conventional in vitro fertilization preimplantation genetic testing for 1 cycle.

**Outcomes:**

Cytogenetic examination showed karyotype 45, X, del (X) del (p11, 2). Ultrasonic examination showed uneven endometrium thickness and a full-stage cystic dilation echo. After 1 cycle of in vitro fertilization treatment, 4 eggs were obtained without forming an available embryo.

**Lessons:**

To our knowledge, the present case is the first report of a patient with TS with Xp deletions and ultrasound imaging endometrial abnormalities. Our findings expand the phenotypic spectrum of TS and may provide a reference for other clinicians.

## 1 Introduction

Turner syndrome (TS), also known as congenital ovarian hypoplasia, is among the most common sexual chromosomal abnormalities in women, with a prevalence of 1/2500 in female live births.^[1]^ TS is caused by partial or complete loss and structural abnormalities of the X chromosome (partial/full or structural distortion; the karyotype may be chimeric or non-chimeric). The most common type of karyotype is the 45, XO, accounting for >60% of cases. Other karyotypes include 45, X / 46, XX; 45, X / 47, XXX; 45, X / 46, XX / 47, XXX; 45, X / 46, X, i (Xq); 45, X / 46, XY; 46, X, del (Xp); 45, X/46, X, del (Xp); 46, X, i (Xp); 46, X, del (Xq); and 45, X /46, X, del (Xq). In our case (Chen XX), the p-arm portion of the X chromosome was deleted, and the karyotype was 45, X, del (X) del (p11, 2). Clinical manifestations of TS vary significantly. Typical features include short stature, hypogonadal hypoplasia, and lymphedema. Commonly, patients manifest only short stature and premature ovarian failure. Studies have indicated that the genes for physical and cognitive features lie on Xp, whereas those for ovarian function are present on both Xp and Xq.^[2]^ According to the 2017 International Turner Syndrome Guidelines with specific classification and incidence reports,^[3]^ patients with TS often present with cardiovascular disease, endocrine, kidney, reproduction, autoimmunity, hearing, visual, and/or psychosocial abnormalities, however, endometrial abnormalities are rarely reported. Herein, we report a case of a patient, whose endometrial thickness was up to 27 mm, and there was a full cystic echo. This case report raises the question whether the relationship between endometrial abnormalities and the genetic etiology of TS requires further study to provide a basis for TS’ clinical treatment.

## 2 Case presentation

**Figure 1:**
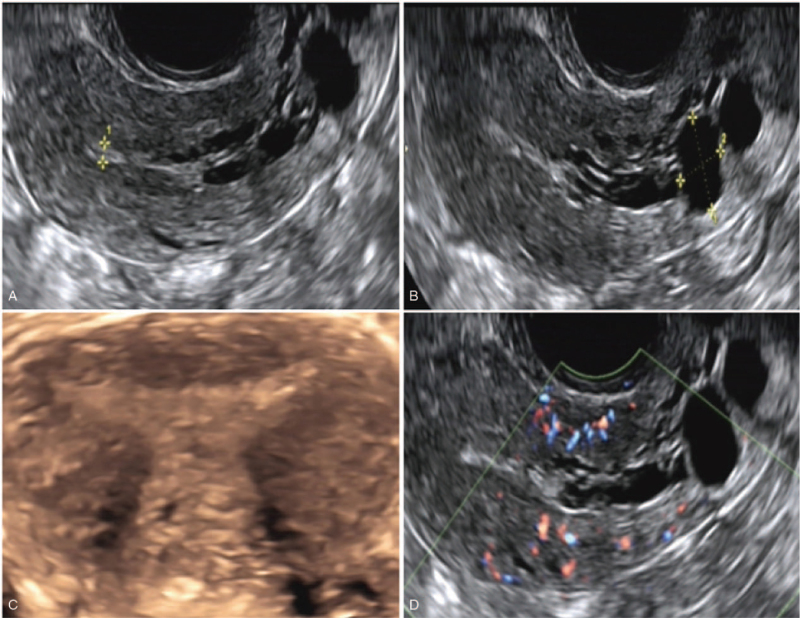
Vaginal ultrasound evaluation on the 16^th^ day of menstruation: ROF: 19.2 mm, LOF: 3 mm, and average intima thickness: 8.8 mm; endometrial 3D color Doppler: multiple cystic echoes in the middle and lower endometrium. LOF = left ovary follicle, ROF = right ovary follicle.

We report the case of a 26-year-old female patient from Fujian, China, who came to our hospital for “living together for 3 years after marriage, no contraception without pregnancy” G0P0. She was born of a non-consanguineous marriage. The age of menarche was 12 years, and she had a menstrual cycle lasting 30 days; her menstrual period lasted for 7 days, and the amount of menstruation was low. The patient was 149-cm tall, weighed 62 kg, and had a body mass index of 27.9 kg/m^2^. Tanner staging was breast 4 and pubic hair 4; she had a poorly developed left labia minora and a smooth vagina. Her bone age was approximately 18 years old, and her height for bone age was <3rd percentile. Biochemical examination: triglycerides 1.99 mmol/L, total cholesterol 5.44 mmol/L, fasting blood glucose 4.19 mmol/L, and serum insulin 9.59 mu/L; the patient's intelligence was normal, and there was no abnormality in the liver, gallbladder, pancreas, spleen, and kidney on ultrasonography. On the 3^rd^ day of menstruation evaluation, her basic hormone levels were as follows: follicle-stimulating hormone, 10.16 mIU/mL (reference range: 1.5–10 mIU/mL before ovulation; 8–20 mIU/mL ovulation period; and 2–10 mIU/mL after ovulation), luteinizing hormone, 5.47 mIU/mL (non-ovulation period: 5–25 mIU/mL; ovulation period: 30–100 mIU/mL; and after ovulation: 4–10 mIU/mL), estrogen, <20 pg/mL (reference range: 48–521 pmol/L before ovulation; 70–1835 pmol/L ovulation period; and 272–793 pmol/L after ovulation), progesterone, 0.21 ng/mL (reference range: usually >15 ug/L), prolactin, 16.08 ng/mL (non-lactation, normal value is 0.08–0.92 nmol/L), testosterone 0.53 ng/mL (follicular phase <1.4 nmol/L; ovulation period <2.1 nmol/L; luteal phase <1.7 nmol/L; and postmenopausal <1.2 nmol/L), and anti-Mullerian hormone, 0.37 ng/mL (reference range: 2–8 ng/mL). The average follicle count was 4, with a size of 6.6 mm, 4 mm, 3.5 mm, and 3 mm; uterus size, 38 × 29 × 28 mm; right ovary size, 18 × 11 mm; and left ovary size, 21 × 11 mm. On the 10^th^ day of menstruation, vaginal ultrasound evaluation showed a right ovary follicle (ROF) 9 mm and a left ovary follicle (LOF), 4 mm; and mean endometrial thickness, 6.6 mm, with unclear morphology and several echo-free zones in almost the whole endometrial section; the maximum was 13 × 9 mm. The endometrial thickness near the uterus bottom was 27 mm, with an unclear morphology. Considering the special conditions of the patient's endometrium, we continued to monitor follicular and intimal development. Vaginal ultrasound evaluation on the 16^th^ day of menstruation showed a ROF, 19.2 mm; LOF, 3 mm; and average thickness of the intima, 8.8 mm; an endometrial 3D color Doppler evidenced multiple cystic echoes in the middle and lower endometrium (Fig. 1).

**Figure 2:**
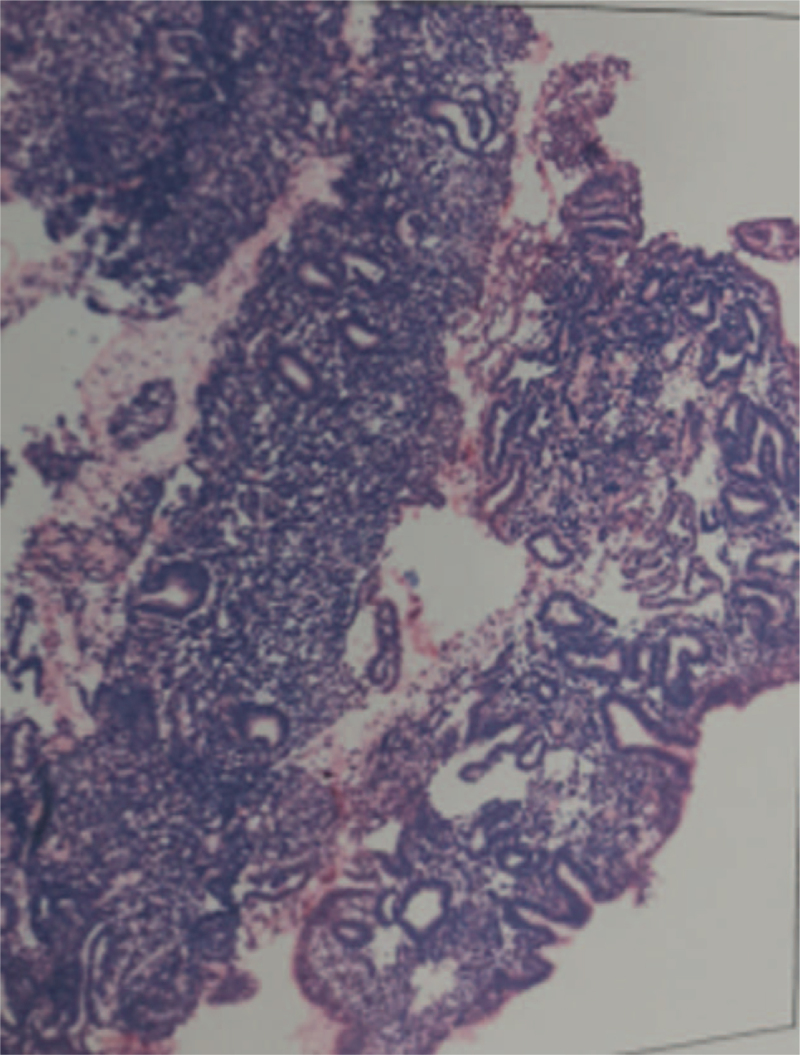
Postoperative pathology results: a small amount of broken endometrial tissue and proliferative endometrium.

The dominant follicle on the right side disappeared on the 18^th^ day of the cycle, and the endometrial thickness was 9.5 mm. We administered luteal progesterone (Abbott Biologicals BV) 1 tablet orally twice a day for 14 days. The patient returned for ultrasound on the 16^th^ day of the second menstrual period. Measurements showed a ROF, 17.9 mm × 1, LOF, 3 mm × 1, and average intimal thickness, 6.8 mm, 5.1 mm at the uterus bottom, with the same morphology as in the previous test with luteinizing hormone 7.59 mIU/mL, estrogen 107 pg/mL, and progesterone 0.32 ng/mL. The patient was hospitalized for “hysteroscopy + treatment.” During surgery, the uterine cavity was normal, the endometrium was flat, and the uterine cavity adhesions were excluded during surgery. Postoperative pathology results were as follows: a small amount of broken endometrial tissue and proliferative endometrium (Fig. 2).

**Figure 3:**
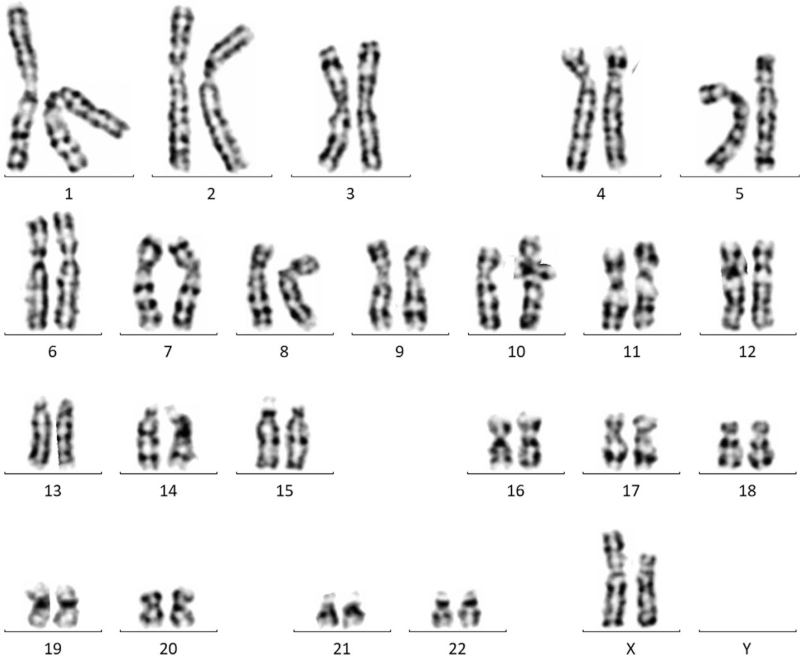
The fertility in patients with Turner syndrome and karyotype 45, X, del(X) del (p11, 2) is low.

Cytogenetic examination revealed a karyotype 45, X, del (X) del (p11, 2) (Fig. 3). The patient was treated with 1 cycle of preimplantation genetic diagnosis-assisted pregnancy therapy. Although 4 eggs were obtained from the patient, no embryos were available. This study fully complied with the tenets of the Declaration of Helsinki and was approved by the Ethics Board of the Women's and Children's Hospital affiliated to Xiamen University, China. Informed consent was obtained from the subject's parents before testing.

## 3 Discussion

TS is a common genetic disease caused by partial or total sex chromosome deletions. The prevalence of TS in 2000 was approximately 1/2500 female births. Currently, the possible mechanisms of TS are: chromosome aneuploidy may induce autoimmune diseases and the X chromosome may contain several autoimmune disease-related genes. When the X-chromosome haplotype dose is insufficient, the risk of autoimmune diseases may increase. According to the 2017 International Turner Guide,^[4]^ 46, Xi (Xq) and 46, X, idci (Xp), that is, the Xq chromosome of the equal arm and the Xp chromosome of the peer double-centromere point account for approximately 10% of the abnormalities in TS. This usually leads to ovarian failure involving Xq13-q26.^[5]^ Female Xp deletion is a typical cause of short stature and ovarian failure. Other female Xp deletions include del(X) (p21.1, p22.1, or p22.2).^[6,7]^

In our case, the patient had a missing Xp11.2, which is a rare type of Xp deletion. The patient also had primary ovarian insufficiency and atypical short stature. The patient's appearance, ability to communicate, hearing, vision, and intelligence were normal, but her body was short with a webbed neck. Although ovarian insufficiency was difficult to diagnose, ovarian dysfunction was present. It is rare that a patient presents with endometrial cystic dilatation in almost a whole section; however, in the current case, the patient's hysteroscopic pathology was normal (maximum 1.32 × 0.95 cm)and unexplained endometrial thickening was observed (maximum 2.7 cm). Its 3D presentation was particularly distinctive, as confirmed by ultrasound experts. Here, we discuss the patient's characteristics from 2 aspects: genetic traits and endometrial specificity.

Some studies suggest that short stature and ovarian failure are the most commonly observed clinical features in patients with TS. However, the genetic mechanism of different TS types and the correlation between genotype and phenotype remain unclear. Studies have shown that^[8]^ the short stature homeobox gene located in the quasi-autosomal region of the short arm of the X and Y chromosomes is almost 100% heterozygous in patients with TS, and short stature could be caused by deletion of the short stature homeobox-encoding gene located on the short arm of the X chromosome.^[9]^ Ovarian dysplasia and infertility are more associated with long-armed X chromosome-arranged monomeric units or deletions. 46, X, idic (Xp) and 46, XX, del (Xq) patients with short arm retention of the X chromosome and long arm loss could have poor ovarian function and even primary amenorrhea. In this case, the patient's karyotype was 45, X, del (X) del (p11, 2), which is a type of p-arm of the X chromosome. Poor ovarian function and short stature were consistent with typical karyotype clinical features. Many studies have reported that women with primary amenorrhea have chromosomal translocation and gonadal dysplasia.^[10]^ X-chromosome abnormalities and translocations and autosomal malformations are more common.^[11]^ In addition, the ovarian function genes are thought to be located on the X chromosome's short and long arms. The short arm of the proximal region of the X chromosome (near the centromere) is more likely to be closely related to ovarian function, including the ubiquitin-specific protease 9 (USP9X)^[12]^ and bone morphogenetic protein 15 (BMP15),^[13]^ located on the short arms, Xp11.4 and Xp11.2, in the proximal region of the X chromosome, respectively. TS patients with distal (near telomere) short-arm-missing X chromosomes tend to retain certain ovarian functions.^[14]^ The insufficiency of X-staining monomers has always been a focus of attention. Candidate gene lists for primary amenorrhea at chromosome breakpoints are listed in Table 1. In addition, according to the latest research on TS epigenetics,^[15]^ KDM6A is an important gene for X chromosome methylation in TS.

**Table 1:** The list of putative candidate genes located at the breakpoint and implicated in the aethology of primary amenorrhea.

Gene	Function
8q24	
NDGRI N-myc downstream regulated genes	Encodes proteins responsible for p53 mediated caspase activation and apoptosis. Involved in stress response hormone response, cell growth and development
PIWiL2:Piwi like protein 2	Involved in development and maintenance of germline stem cells
EERD Eukaryotic elongation factor 1 delta	Encodes a submit of elongation factor 1 complex responsible for enzymatic delivery of aminoacyl tRNA's to ribosomes
PARP30	Regulates gene transcription by altering chromatin organization
poly (ADP ribose)polymerase	
RHPN1-AS1 Rhophilin Rho GTPase Binding	Interact with cytoskeletal component upon Rho binding and helps in signaling of her molecules
protein 1	
ZNF16 Zinc Finger Protein 16	Transcriptional activator, promotes cell proliferation, differentiation of erythroid and megakaryotic cells inhibitor of cell apoptosis
ZNF16 Zinc Finger Protein 7	Involved in transcriptional regulation
5q14	
ED1L3 EGF like repeats and discoidin	Plays an important role in mediating angogenesis and important in vessel wall remodeling and development
domains 3	
NR2F1 Nuclear receptor subfamily 2, groupp	Nuclear hormone receptor and transcriptional regulator
F member1	

To date, there have been no reports of TS patients with endometrial ultrasound abnormalities, except for 1 case of a TS patient with endometrial cancer, where the final pathological diagnosis was endometrial adenocarcinoma.^[16]^ In a previous report on the chimeric karyotype of 45, XO for TS, only 16% of patients had spontaneous menstruation and 8 of 10 (80%) TS patients (*P* < .0001) contracted endometrial cancer in the absence of conventional hormonal therapy (t < 0.0001).^[16]^ Regarding cystic endometrium dilatation,^[17]^ 1 study reported that endometrial gland expansion was approximately 99.2% independent of endometrial atypia. In addition, in 2995 women with endometrial disease, Felix et al^[18]^ reported a cutoff value for ultrasound assessment of endometrial hyperplasia, endometrial cancer, and normal endometrium of 8 mm. Sudden endometrial hyperplasia is often associated with excess estrogen production. The endometrium thickness in this case was 27 mm, significantly higher than that before normal ovulation. Endometrial cancer was ruled out, and there was no significant increase in estrogen; hence, we speculated that the endometrial thickness may also be related to endometrium receptors. Simultaneously, although in this case we could not determine the cause of the cystic dilation of the entire endometrium, this process may return to normal at certain hormone levels.

## 4 Conclusions

In conclusion, we encountered a case of TS with Xp deletions with primary ovarian insufficiency and endometrial ultrasound imaging abnormalities. Further research on the determinant factors and X chromosome genes related to growth, ovarian function, and autoimmunity is warranted. Our findings enrich the phenotypic spectrum of TS and are expected to provide a reference for clinical applications.

## Author contributions

LL and MLB carried out research design, experiments, analyzed data, explained the results, and drafted manuscripts. SYY and HLL participated in the data collection. SZY and ZY participated in the design and interpretation of the research results. GHJ and HXM were responsible for ultrasound examination and interpretation of the results. HH was involved in the experiment. HYR, LP, and CJ helped to design the work and revised the final version of the manuscript.

**Conceptualization:** Lei Liang, Libin Mei, Haijie Gao, Hui Huang.

**Data curation:** Lei Liang.

**Formal analysis:** Lei Liang, Libin Mei.

**Funding acquisition:** Libin Mei, Yanru Huang.

**Investigation:** Lei Liang.

**Methodology:** Lei Liang, Libin Mei.

**Project administration:** Libin Mei, Lingling Huang.

**Resources:** Xuemei He.

**Software:** Yingying Shi.

**Supervision:** Zhiying Su, Ping Li.

**Validation:** Yu Zeng, Jing Chen.

**Visualization:** Haijie Gao, Ping Li.

**Writing – original draft:** Lei Liang, Jing Chen.

**Writing – review & editing:** Lei Liang, Yanru Huang.
